# Sexual dysfunction among people with mental illness attending follow-up treatment at a tertiary hospital, Jimma University Medical Center: A cross-sectional study

**DOI:** 10.3389/fpsyt.2022.999922

**Published:** 2022-11-17

**Authors:** Jerusalem Sewalem, Chalachew Kassaw, Tamrat Anbesaw

**Affiliations:** ^1^Department of Psychiatry, College of Medicine and Health Science, Dilla University, Dilla, Ethiopia; ^2^Department of Psychiatry, College of Medicine and Health Science, Wollo University, Dessie, Ethiopia

**Keywords:** sexual dysfunctions, mental illness, psychiatry clinic, follow up treatment, Ethiopia

## Abstract

**Background:**

Sexual function is a complex behavior influenced by several factors that can result in dysfunction. It is highly prevalent among patients with mental illness who are on psychotropic medications. Assessing those patients has paramount importance for appropriate intervention to take place.

**Methods:**

This study was a facility-based cross-sectional study design conducted from 1 March to 30 June 2022. A Sexual Functioning Questionnaire (CSFQ-14) was used to assess sexual dysfunctions. Data were analyzed using SPSS version 21 software. Logistic regression analysis was performed to assess the association between dependent and independent variables. Independent variables with a *p*-value < 0.05 were taken as statistically significant with sexual dysfunction.

**Results:**

The prevalence of sexual dysfunction was 45.4 % among respondents. The presence of chronic medical illness, being on typical antipsychotic treatment, being on psychiatric treatment for 24 months and more, moderate level of alcohol use, and being aged 35 and above were significantly associated with sexual dysfunction.

**Conclusion:**

The prevalence of sexual dysfunction among people with mental illness is high. Therefore, the clinician needs to routinely enquire about sexual symptoms during follow-up treatment and give appropriate interventions with special attention to patients with chronic medical conditions and patients taking antipsychotics and psychotropic drugs for a long period of time.

## Background

According to the World Health Organization, sexual health is a state of physical, emotional, mental, and social well-being concerning sexuality. It is not merely the absence of disease, dysfunction, or infirmity and sequel. Sexual health requires a positive and respectful approach to sexuality and sexual relationships. In addition, it includes the possibility of having pleasurable and safe sexual experiences free of coercion, discrimination, and violence. For sexual health to be attained and maintained, the sexual rights of all persons must be respected, protected, and fulfilled (1). Sexuality assessment and records started during the time of Hippocrates. It has been an increasing concern in recent years (2).

Global studies on sexual attitudes and behaviors state that lack of interest and inability to reach orgasm during sexual intercourse was the most common sexual problem across the world. It ranges from 26 to 43% and 18 to 41%, respectively (3). Sexual complaints are common problems among the general population in the United States. Its prevalence is estimated to be 31% in males and 43% in females (4, 5).

From a psychiatric perspective, sexual dysfunctions can be considered as an alteration in one or more phases of the sexual response cycle, such as sexual desire, sexual excitement, orgasm, or climax and resolution (6, 7). Studies show that sexual dysfunction might be related to primary or secondary mental illness, psychotropic medications, psychosocial issues, and the use of substances (8, 9).

Results of epidemiological and clinical studies indicate that depression, anxiety, and medications had approximately a 30 to 70% impact on sexual function (9–11). Adult participants who took SSRIs had about 65–94% risk of sexual impairment across different subscales of CSFQ-14 (4, 12, 13).

Patients with chronic mental illnesses like schizophrenia are vulnerable to sexual dysfunction. The study showed that around half of schizophrenic patients on antipsychotic medication had sexual dysfunction due to the nature of the illness (14, 15). Substance use is also a growing problem that leads to sexual dysfunction. Among alcohol users, 26% had sexual dysfunction. Of these figures, males had about 72% of dysfunction (16). Likewise, among smokers smoking more than 20 cigarettes per day, 50% of them had sexual dysfunction (14, 17–19).

Inversely, sexual dysfunction causes anxiety, sadness, depression, low self-confidence, low self-esteem, marital tension, guilt, anger, and frustration in a relationship leading to the deterioration of quality of life and causing non-adherence to medication (18–20). An Ethiopian study reported that sexual dysfunction among patients with pelvic floor disorder was 47% (21), epilepsy, 63.9% (22), mental illness, 73.02% (23), and diabetes mellitus 69.9% (24).

Despite the high prevalence of sexual dysfunction, particularly among patients taking psychotropic medications, most sufferers do not seek help due to feelings of embarrassment (20). Additionally, the majority of professionals give it little emphasis and do not enquire about the sexual health of their clients (or sexual dysfunctions) in their routine clinical setting (25, 26). Furthermore, there is little evidence in Ethiopia, particularly in the study area. Therefore, the current study was conducted to estimate the level of sexual dysfunction among people with mental illness attending follow-up treatment. It serves to help professionals providing care to these groups of people to provide them with appropriate interventions for the problem.

## Methods and materials

### Study design and study period

An institutional-based cross-sectional study was conducted from 1 March to 30 June 2022.

### Study setting

The study was conducted at the psychiatric clinic at Jimma University Medical Center (JUMC). The JUMC is the only teaching and referral hospital in southwest Ethiopia providing medical services, including inpatient and outpatient psychiatry treatment. On average, each month more than one thousand people with mental illnesses receive outpatient psychiatric care in the clinic.

### Population

The inclusion criteria for this study were: age 18 years and above, currently in a good mental state, and attending outpatient follow-up treatment in the clinic during the data collection time. The exclusion criteria were respondents with acute exacerbation of illness and cognitive impairment.

### Sample size determination and sampling technique

The sample size was determined using the single population proportion formula at a 95% confidence interval, 5% marginal error, 50% population proportion, and a 10% non-response rate. The final sample of the study was 423. A consecutive sampling technique was employed to select study participants during the data collection time.

### Variables

In the current study, the outcome variable was the sexual dysfunction of males and females. Socio-demographic and economic factors, history of substance use, psychiatric diagnosis, medication-related factor (treatment duration, type of medication), and history of chronic medical illness were the independent variables.

### Data collection method and instrument

The data were collected using structured questionnaires through face-to-face interviews. Sexual dysfunction was assessed by using the sexual functioning questionnaire (CSFQ-14), a short form that is a more amendable and less time-consuming tool commonly used in clinical practice. The CSFQ-14 is a valuable new tool for detecting the presence of male and female sexual disorders and identifying the specific components of sexual functions affected (desire, arousal, orgasm, or dyspareunia). It has a good construct validity and internal reliability (Cronbach's alpha = 0.90) (27). The questionnaire has five sections: sexual desire frequency, sexual desire interest, sexual pleasure, sexual arousal, and orgasm. Additional questions ascertain the degree to which sexual functioning has changed over time. It also assesses extensive changes and measures the nature and underlying cause of these changes (4). The instrument evaluates three phases of the sexual response cycle: desire (items 2–6), arousal (items 7–9), and orgasm (11–13). Items 10 and 14 are not specific to a phase of the sexual response cycle. Items are rated by the respondent using a 5-point Likert scale of frequency (1 = never to 5 = every day/always) or intensity (1 = nothing to 5 = very much), except for items 10 and 14 for which 1 = every day/always and 5 = never. The total CSFQ-14 score ranges from 14 to 70, and higher scores reflect better sexual functioning, but total scores ≤ 47 for males and ≤ 41 for females suggest global sexual dysfunction. The CSFQ is usually used to screen patients' sexual dysfunction at baseline and after 6 months of taking medication in the psychiatric clinic (28).

This questionnaire was translated into the local languages Amharic and Afan Oromo and back-translated to English, to ensure its consistency. Finally, the Amharic and Afan Oromo version questionnaire was administered to collect data.

### Data quality control

3 days of training were provided for data collectors on how to collect data, data collection methods, tools, and how to handle ethical issues. Regular monitoring was undertaken by the supervisor and the principal investigator to ensure that all necessary data was properly collected. During data collection, filled questionnaires were checked daily for completeness and consistency by the supervisors. Four BSc psychiatry nurses and two BSc clinical nurses served as data collectors. Two health professionals with experience in previous supervision were used as supervisors. The patients were individually asked to answer the questionnaires in selected interview rooms. A pre-test was conducted in the same study setting before the actual data collection to identify potential problems with data collection tools and to check the performance of the data collectors. Data that were collected in the pre-test were not included in the analysis as part of the main study.

### Data processing and analysis

Once all necessary data were obtained, it was checked for completeness and entered using Epi Data manager version 4.0.2 and exported to SPSS version 21 for analysis. Socio-demographic characteristics of participants were described using simple descriptive statistics such as mean, percentage, frequency, and standard deviation. In addition, binary and multiple logistic regressions were conducted to explore association and to identify independently associated variables. A *P*-value < 0.05 was taken to declare a statistically significant association. The strength of association was measured using an odds ratio and 95% confidence level.

## Results

### Socio-demographic characteristics

A total of 421 patients participated in this study with a response rate of 99.5%. Of the total number of participants, 76.5% (*n* = 322) were males. The mean age of the respondents was 34.53 years with SD ± 11.25. The majority (56.8%, *n* = 239) of them were Muslims and ethnically Oromo (71.7%, *n* = 302). Regarding the educational status of respondents, 30.6% (*n* = 129) had attended primary school and 58.7% (*n* = 247) of respondents resided in urban settings. More than half (52.4%, *n* = 220) were married. Among the respondents, 61.3% (*n* = 258) had private businesses and the highest number of respondents earned 50–400 Ethiopian birr per month (27.1%, *n* = 114) (Table 1).

**Table 1:** Socio-demographic characteristics of respondents attending follow-up treatment at the University Medical Center Psychiatry Clinic, Jimma, southwest Ethiopia, 2022 (*N* = 421).

**Variables**	**Categories**	**Frequency**	**Percent**
Sex	Male	322	76.5
	Female	99	23.5
Age	18–27	122	29.0
	28–33	128	30.4
	34–39	66	15.7
	40 and above	105	24.9
Educational status	No education	79	18.8
	Primary	129	30.6
	Secondary	104	24.7
	Tertiary education level	109	25.9
Marital status	Single	168	39.8
	Married	220	52.4
	Divorced and windowed	33	7.8
Religion	Orthodox Christian	122	29.0
	Muslim	239	56.8
	Protestant	58	13.8
	Catolic	2	0.5
Ocupation	Unemployed	52	12.3
	Private business	258	59.2
	Governmental employ	102	26.3
	Non-governmental employ	9	2.1
Ethnicity	Oromo	302	71.7
	Amhara	58	13.8
	Tigre	17	4.0
	Dawro	34	8.1
	Other^**^	10	2.4
Living place	Urban	247	58.7
	Rural	174	41.3
Monthly income	50–400	114	27.1
	401–800	107	25.4
	801–1,425	95	22.6
	< =1,426	105	24.9

^**^Silte and gurage in ethinicity group.

### Clinical diagnosis of patients with mental illness

Among the study participants, 41.8% (*n* = 176) were diagnosed with schizophrenia. Out of the total participants, the duration of illness was 2-5 years for 35.4% (*n* = 149). Of all participants, 8.1% (*n* = 34) had a medical illness and 79.4% (*n* = 27) of them were using medication for their illness. The majority of the participants (55.9%, *n* = 19) had hypertension, and 1.7% (*n* = 7) had major surgery on their genital area. About 2.6% (*n* = 11) of the participants had a history of rape in their lifetime (Table 2).

**Table 2:** Psychiatric and medical clinical diagnosis among respondents attending follow-up treatment in the University Medical Center Psychiatry Clinic, Jimma, southwest Ethiopia, 2022 (*N* = 421).

**Variable**	**Categories**	**Frequency**	**Percent**
Pychaitery illness	Schizophrenia	176	41.8
	Major depression disorder	143	34.0
	Bipolar disorder	68	16.2
	Other psychateric illness^*^	34	8.1
Duration of mental illness Mean (SD = ±45.0)	Below 60 month	210	49.9
	60 month and above	211	50.1
Duration of current psychiatry medication Mean (SD = ±18.1)	Below 24 month	188	44.7
	Above 24 and month	233	55.3
Known chronic medical illnesses	Yes	34	8.1
	No	387	91.9
Diagnosied chronic medical illness	Hypertension	19	55.9
	Diabitic malites	9	26.5
	Human Immunodeficiency Virus	2	5.9
	Hyperthyroid	3	8.8
	Epilepsy	1	2.9
Use of medications for chronic medicalillnesses	Yes	27	79.4
	No	7	20.6

^*^Anxiety disorder, somatic disorder.

### Prevalence of sexual dysfunction among mentally ill patients

Sexual dysfunction among patients who participated in the study was 45.4% (*n* = 191). After reviewing the independent variables (sex, marital status, educational status, occupation, religion, residence, and type of psychiatry illness), participants who had known diagnosed chronic medical illness were found to be 4.90 times more likely to have sexual dysfunction than those participants free of diagnosed medical illness (AOR = 4.90, 95% CI = 1.65–14.48). Those aged 35 and above were more affected by sexual dysfunction (AOR = 1.91, 95% CI = 1.12–3.26) than participants aged below 35. The chance of sexual dysfunction among participants who were taking typical antipsychotic medications was 2.27 times (AOR = 2.27, 95% CI = 1.26–4.09) higher than those not under typical antipsychotic medication. Participants who were currently on psychotropic medications for 24 months and above were 1.74 times (AOR = 1.74, 95% CI = 1.03–2.92) more likely to have sexual dysfunction than those who were under medication for < 24 months. Those who took moderate levels of alcohol were 7.36 times (AOR = 7.36, 95% CI = 1.41–38.32) more affected by sexual dysfunction than participants who consumed low levels of alcohol (Table 3).

**Table 3:** Bivariate and multivariate analyses of factors associated with sexual dysfunction among respondents attending follow-up treatment at the Jimma University Medical Center Psychiatry Clinic, Jimma, southwest Ethiopia, 2022, (*N* = 421).

**Variable**	**Crude OR(95%CI)**	**AOR(95%CI)**
**Age MEAN(SD** **=** **11.25)**		
< 35year	1	1
≥35 year	2.12(1.42–3.17)^**^	1.91(1.12–3.26)^**^
**Diagnosed chronic medical illness**		
No	1	1
Yes	3.16(1.47–6.79)^**^	4.90(1.65–14.48)^**^
**Duration of current medication**		
< 24 months	1	1
≥24 months	1.29(0.88–1.90)	1.74(1.03–2.92)^**^
**Taking typical antipsychotic currently**		
No	1	1
Yes	2.27(1.52–3.40)^**^	2.27(1.26–4.09)^**^
**Level of alcohol use**		
Low	1	1
Moderat	3.62(0.74–17.76)	7.36(1.41–38.32)^**^
High risk	0.46(0.14–1.53)	0.45(0.13–1.63)

^**^P value < 0.01.

## Discussion

The prevalence of sexual dysfunction among the study participants in this study was 45.4% with a CI (of 40–50%). The prevalence was similar to a study conducted in India (29), but, lower than a study from the United States (30). The difference could be due to variation in the study population in which the latter used only schizophrenia and schizoaffective patients, which suggests that the higher prevalence might be due to the psychopathology of illness and its medication (4, 5).

Regarding psychotropic medication, participants who were on typical antipsychotic medication were two times more likely affected by sexual dysfunction as compared to those participants free of typical antipsychotics. This finding was supported by a study done on patients taking conventional antipsychotic medication in which 45% of participants developed sexual dysfunction (5). It could be directly related to the mechanism of action that increases prolactin levels in the tuberoinfundibular pathway among typical antipsychotics.

In this study, it was observed that factors like diagnosed chronic medical illnesses increased the risk of sexual dysfunction five times. This finding was supported by a study done in Canada on risk factors for sexual dysfunction which found that diabetes, heart disease, urinary tract disorders, and chronic illnesses were significant risk factors for sexual dysfunction (31). It might be perhaps people with mental illnesses have a higher rate of smoking, alcohol consumption, poor diet, and lack of exercise (32). It could also be due to untreated medical illness in psychiatric patients causing an additional effect on sexual health.

In this study, participants who were on current psychotropic medications for 24 months and above had two times more sexual dysfunction than those who were on psychotropic medications below 24 months. This finding was supported by a study done on the prevalence of sexual dysfunction among patients with mental illness and receiving psychotropic medication, and a study on sexual dysfunction mechanisms and management which suggested that a high prevalence of sexual dysfunctions occur in those patients receiving antipsychotics treatment for a longer duration and the effects depend on the length of exposure to medication (33).

Participants over the age of 35 had roughly a 2-fold higher risk of having sexual dysfunction than participants under the age of 35. This could be explained by physical ailments brought on by aging and psychological aspects that lead to sexual issues. Even in the general population, about one-third of older adults have at least one sexual function complaint (15). The current study's participants also had mental illnesses, which can have an impact on sexual function on their own. These factors may have interacted to cause an increase in sexual dysfunction with age.

### Limitations of the study

The main limitation of this study was its cross-sectional nature which does not show a cause-effect relationship between the outcome and independent variables. Undiagnosed medical conditions were excluded from the analysis of this investigation, which could be a potential weakness of this study. It was therefore thought to be one of the recognized confounders in the relationship of findings. The main limitations of this study were that the CSFQ-14 screening instrument does not identify the length of sexual dysfunction, and social desirability bias because the study covers more delicate subjects. For future studies, it is better if factors such as the onset of the first sexual experience, dosage of each psychotropic medication, history of masturbation, and watching pornography were included for a better understanding of sexual dysfunction.

## Conclusion

Sexual dysfunction was prevalent among those who had mental illnesses. Since it enhances the likelihood of having a problem, mental health practitioners who treat people with mental illness should regularly examine their patients' sexual health.

## Data availability statement

The raw data supporting the conclusions of this article will be made available by the authors, without undue reservation.

## Ethics statement

The studies involving human participants were reviewed and approved by Jimma University. The patients/participants provided their written informed consent to participate in this study.

## Author contributions

All authors listed have made a substantial, direct, and intellectual contribution to the work and approved it for publication.

## Funding

This study was funded by Jimma University College of Medicine and Health Science.

## Conflict of interest

The authors declare that the research was conducted in the absence of any commercial or financial relationships that could be construed as a potential conflict of interest.

## Publisher's note

All claims expressed in this article are solely those of the authors and do not necessarily represent those of their affiliated organizations, or those of the publisher, the editors and the reviewers. Any product that may be evaluated in this article, or claim that may be made by its manufacturer, is not guaranteed or endorsed by the publisher.
